# NucBase, an easy to use read mapper for small RNAs

**DOI:** 10.1186/1759-8753-4-1

**Published:** 2013-01-01

**Authors:** Jeremy Dufourt, Pierre Pouchin, Pierre Peyret, Emilie Brasset, Chantal Vaury

**Affiliations:** 1 Clermont Université, Université d’Auvergne, Laboratoire GReD, BP 38, F-63001 Clermont-Ferrand, France; 2 Inserm U 1103, F-63001, Clermont-Ferrand, France; 3 CNRS, UMR 6293, F-63001, Clermont-Ferrand, France; 4 CHRU, F-63001, Clermont-Ferrand, France; 5 EA4678, Université d’Auvergne, F-63001, Clermont-Ferrand, France

**Keywords:** Alignment, Short reads

## Abstract

**Background:**

High-throughput deep-sequencing technology has generated an unprecedented number of expressed sequence reads that offer the opportunity to get insight into biological systems. Several databases report the sequence of small regulatory RNAs which play a prominent role in the control of transposable elements (TE). However, the huge amount of data reported in these databases remains mostly unexplored because the available tools are hard for biologists to use.

**Results:**

Here we report NucBase, a new program designed to make an exhaustive search for sequence matches and to align short sequence reads from large nucleic acid databases to genomes or input sequences. NucBase includes a graphical interface which allows biologists to align sequences with ease and immediately visualize matched sequences, their number and their genomic position. NucBase identifies nucleic motives with strict identity to input sequences, and it capably finds candidates with one or several mismatches. It offers the opportunity to identify “core sequences” comprised of a chosen number of consecutive matching nucleotides. This software can be run locally on any Windows, Linux or Mac OS computer with 32-bit architecture compatibility.

**Conclusions:**

Since this software is easy to use and can detect reads that were undetected by other software, we believe that it will be useful for biologists involved in the field of TE silencing by small non-coding RNAs. We hope NucBase will be useful for a larger community of researchers, since it makes exploration of small nucleic sequences in any organism much easier.

## Background

Next-generation sequencing has recently emerged as a powerful technology that provides unprecedented insight into biological systems [1]. With automated sequencing technologies becoming routinely used, the acquisition of millions of sequence reads per experiment leads researchers to the subsequent challenge of data analysis. Many of these data are now made available in databases that provide the scientific community with an invaluable shared resource. The number of databases reporting sequences of non-coding small RNAs has dramatically increased during the past five years and offers tremendous promise for elucidating the role of siRNAs, piRNAs and miRNAs in health and disease [2]. The class of small RNAs called piRNAs is mainly involved in transposable element regulation in the germline cells of many organisms (mice, rats, *Drosophila* and other animal species). These Piwi interacting RNAs (piRNAs) are bound to proteins of the argonaute PIWI family. They recognize and destroy mRNA encoded by transposable elements, preventing their propagation [3-6]. In 2007, the GJ Hannon laboratory published the first large database of piRNAs from *Drosophila* with approximately 17,000 reads [7]. One year later, more than one million reads were reported in a database from deep sequencing piRNAs [8].

Yet, the huge amount of information contained in these databases remains mostly unexplored because the informatics tools currently available are difficult to use. An efficient, easy handling tool allowing small RNA mining and alignment would help to further elucidate the mechanism of mRNA recognition by small RNAs.

Here we introduce NucBase, a new program designed to align short reads from large nucleic acid databases to reference sequences or genomes. Mapping short reads can be achieved using other tools like SOAP [9], BWA [10], Bowtie [11] and vmatch [12], which are very fast, but omit potential matches due to their greedy algorithms. NucBase is an easy-to-use program with a graphical interface. It identifies nucleic motives with strict identity to input sequences and also finds candidates with one or several mismatches. Furthermore, this software can identify partial fragments from a read that match a target sequence. The user is able to select the number of consecutive nucleotides within these partial fragments, called “core sequences,” as well as the number of possible nucleotide mismatches. These “core sequences” can be detected regardless of position within the read. Results are exported in a Generic Feature Format 3 (GFF3) output format, which permits the visualization of read alignments on target sequences and fast annotation on the GBrowse viewer for a better understanding of potential regulations.

### Implementation

NucBase is an easy-to-use program developed in C++ with a Qt graphical interface, whose aim is to align millions of reads on target sequences. For this purpose, it first computes the Burrows-Wheeler Transform (BWT) [13] of the target, as well as its suffix array, through the fast and lightweight LibDivSufSort library (Mory, 2010). These two elements, which form an FM-Index, are then used to find all the positions where there are exact read matches [14,15]. In addition, NucBase can allow a variable number of mismatched characters, which identify reads with imperfect matches [16]. As a third alternative, NucBase offers the possibility to find a partial, consecutive sequence called a “core sequence” from a read. The size, L, of that core sequence is chosen by the user as well as the number of mismatches to introduce. This unique feature of NucBase allows for more flexibility in the search for new piRNA targets.

If we consider a string, S, which ends with a unique character that should be the lowest in the lexicographic order, then, by computing all the cyclic permutations of S and ordering them alphabetically, we obtain the Burrows-Wheeler Transform of S, BWT(S): the last column of this matrix. Moreover, if we call F(S) the first column of the matrix, then the suffix array, SA(S), designates the vector containing the original positions of each character in F(S). A particular property of the transformed sequence is that the n^th^ occurrence of a character in BWT(S) is the n^th^ occurrence of said character in F(S). Therefore, as suggested by Ferragina and Manzini [14], searching for a word of size M in the original string of size N is easy and fast (complexity: O(M)).

We know all of the adjacent positions of the word’s last character in F(S). Since BWT(S) is obtained through cyclic permutations, the characters in BWT(S) are the ones preceding those in F(S). By counting the number of occurrences of the penultimate character in BWT(S) at the specified positions, we can determine its positions in F(S).

**Figure 1:**
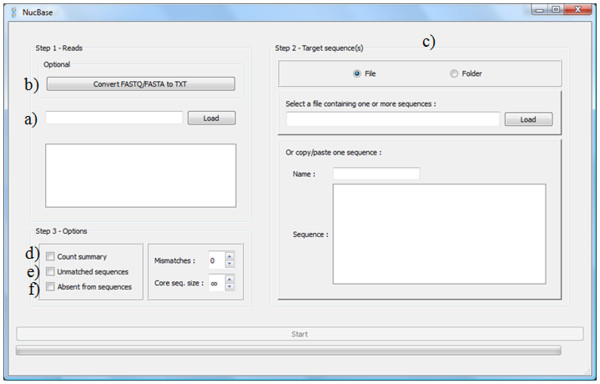
**NucBase GUI**. The NucBase graphical interface allows the user to load a file containing reads (**a)** which can be converted beforehand to the appropriate format (**b**). Then, a folder or a file containing the sequences of interest must be selected (**c**). It is also possible to directly copy and paste a sequence in the main window. Before starting the software, the user can specify several options to generate a global summary table giving the total number of matches for each read (**d**)**,** to export the unmatched sequences (**e**), or to output only non-matching reads (**f**).

**Figure 2:**
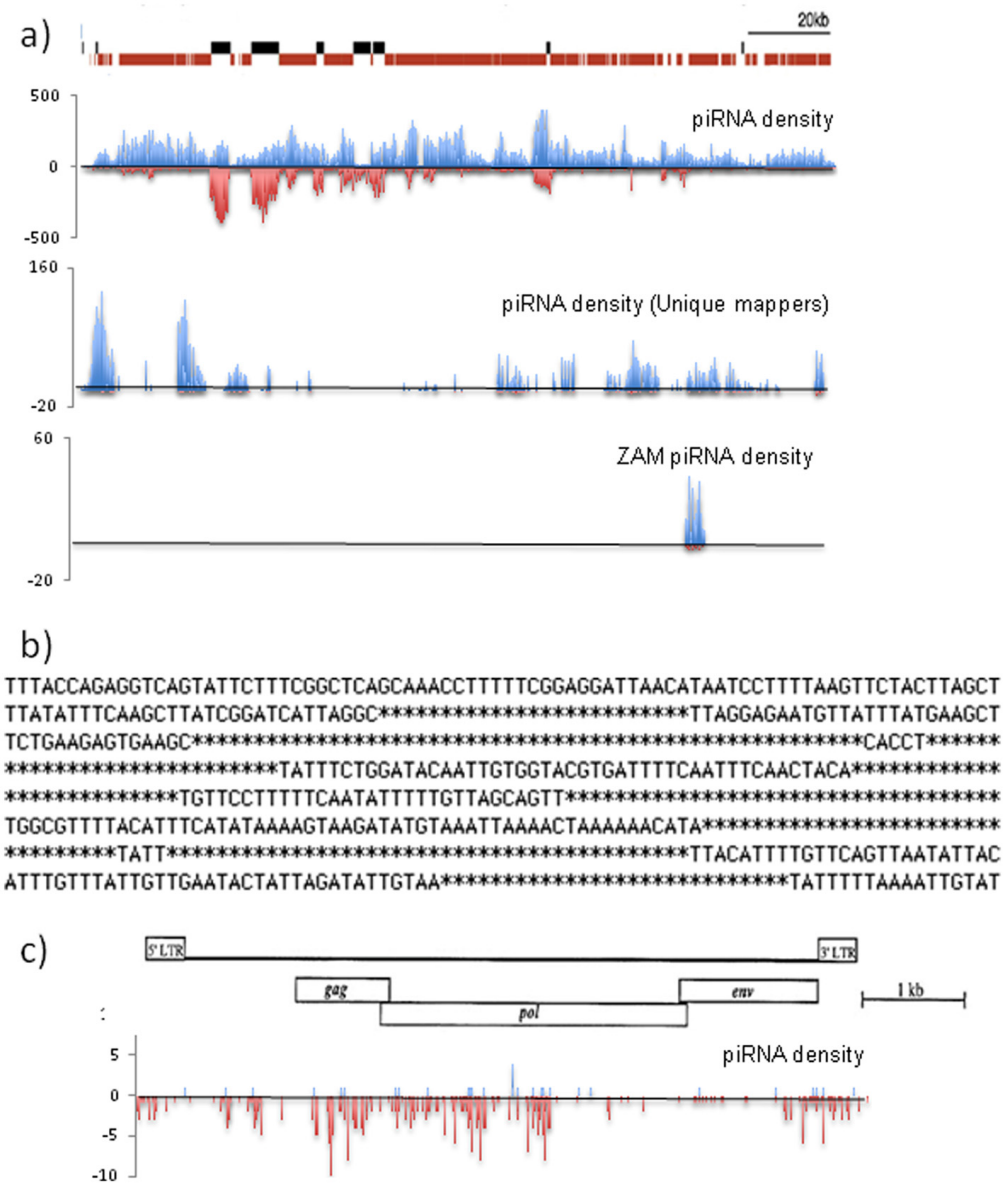
**NucBase identified piRNAs mapping to the *flamenco *locus.** (**a**) Density of annotated transposons of the *flamenco* locus (X:21501186.1684449) (revised from Brennecke *et al.*, 2007) indicating sense (Black) and antisense (red) transposon fragments. Density of all piRNAs, the unique mappers piRNAs and ZAM piRNAs (blue: sense - red: antisense) along the *flamenco* locus from the 2008 Brennecke piRNA database. (**b**) A representative view of a *flamenco* fragment (X:21503826.21504465). Asterisks indicate the location of read matches with the reference *flamenco* sequence. The unmatched sequences are immediately visualized. (**c**) Organization of the *ZAM* element with locations of the different ORFs and piRNAs density along the ZAM sequence.

As an input, NucBase needs a file containing the “reads” (Figure 1a) in a tab-separated values text file-FASTA and FASTQ files can be converted beforehand to this format (Figure 1b)- and the “target sequences”, either in a FASTA/multi-FASTA file or directly pasted into the software main window (Figure 1c). There are two main output formats: General Feature Format (GFF3) and text (same as input). By default, the software will produce two table files containing the number of matches found in the sequence on both strands, as well as a GFF3 file containing the positions. It is also possible to produce a global summary table giving the total number of matches for each read (Figure 1d), as well as a FASTA file containing each target sequence on both strands with an asterisk indicating the location of a read match (Figures 1e and 2). This allows the biologist to immediately visualize matched and unmatched sequences. There are two additional features that depend on the input database file: if the file contains reads with a descriptive line, the output files will indicate the corresponding name; if reads contain degenerate bases, the software will test each possibility. Finally, the software only outputs matching reads by default, but it is possible to also save non-matching reads (Figure 1f).

## Results and discussion

We compared NucBase performance to the very efficient Bowtie alignment program, which is widely used and among the fastest competing open source tools for aligning millions of reads. These tests were run on a Windows Vista x64 workstation with 16 cores and 48 GB of RAM. By mapping w^1118^ ovary piRNAs from the database published by Brennecke *et al.*[8]) (701,853 reads) onto the *Drosophila* genome, we evaluated the speed and sensitivity performance of NucBase compared to Bowtie. Since NucBase always reports all the alignments, we used the corresponding option in Bowtie during these tests. Table 1 reports the results. We found that the speed of Bowtie and NucBase is comparable: NucBase is 2.6 times faster for perfect matches, but 1.7 times slower with 2 mismatches. Speed and memory improvements of NucBase are planned for the future.

**Table 1:** Speed and sensitivity performance of Bowtie and NucBase

	**Bowtie**	**NucBase**
0 mismatch	292 s	109 s
	701,699 reads aligned	701,699 reads aligned
	28,473,348 alignments	28,473,348 alignments
2 mismatches	1,489 s	2,565 s
	701,748 reads aligned	701,748 reads aligned
	94,372,576 alignments	94,541,096 alignments

Mapping of ovarian piRNAs from Brenecke’s database (2007) onto the *Drosophila* genome.

Both programs reported the same number of aligned reads with perfect matches (28,473,348 alignments).The number of alignments slightly differed with two mismatches: 94,541,096 alignments with NucBase and 94,372,576 with Bowtie. One major difference in this test is that Bowtie limits the number of allowed mismatches to three while NucBase has no such limit. It must be pointed out that this characteristic may be useful to biologists working on repeated copies of mobile elements and taking into account active copies and vestiges of ancient insertions.

In addition, NucBase displays two major interesting features that are not shared with Bowtie.

First, alignments involving one or more ambiguous reference characters (IUPAC code) on reads are considered invalid and treated as an A by Bowtie but not by NucBase. If the R character is present in a read, NucBase will align successively A and G, and identify reads with either of the two nucleotides. On the contrary, Bowtie will only test A. Due to this flexibility, short consensus sequences can be mapped appropriately by NucBase but not by Bowtie.

Second, NucBase allows the identification of core sequences regardless of position within the read whereas Bowtie only identifies these sequences (called seeds) if they are positioned at the 5′ end of the read. Mapping Brennecke’s database [7] to the *nanos* gene of *Drosophila*, we searched for a core sequence of 18 nucleotides with one mismatch allowed. The number of reads recovered by NucBase and Bowtie are 1,702 and 501, respectively. This indicates that NucBase allows alignments that are ignored by Bowtie. To go further, we searched for a core sequence recently described in literature. Rouget *et al.* have searched for piRNAs sequenced from early *Drosophila* embryos and presumed capable of targeting the 3′ untranslated region of the gene *nanos* (*nos* 3′UTR) based on their sequence complementarity [17]. Along with Rouget *et al.*, NucBase identified two specific regions located in the 3′-most part of the 3′UTR that could be targeted by piRNAs originating from two transposable elements, 412 and roo (TATATATATATGTGTGTT and AATTGAATAAATATAT). Bowtie only identified one of them (AATTGAATAAATATAT). This difference is due to the fact that Bowtie only aligns contiguous blocks starting at the 5′ end of the reads.

### NucBase use through an example

We made use of Nucbase to characterize piRNAs reported in databases and involved in the silencing of ZAM, a retrotransposon from *Drosophila melanogaster*. The *flamenco* cluster, (*flam*) which encompasses the 180 kb of proximal sequence from the *DIP-1* gene in the pericentromeric region of the X-chromosome is a major piRNA cluster, and is involved in ZAM silencing [3,7].

We used NucBase to further characterize the piRNAs produced by *flam* (Figure 2). All the piRNAs from the 2008 Brennecke database, were mapped to the *flam* sequence (X:21,495,823..21,684,449) from release 5 of the *Drosophila* genome sequence) (Figure 2a, top). Among 701,853 piRNAs from the database, 85,420 matched *flam*. Of the *flam* matching piRNAs, 70,795 are produced from the + genomic strand of *flam*, 28,418 from the - genomic strand and 13,793 from both strands (Figure 2a). NucBase offers the possibility of aligning these piRNAs along the *flam* sequence and visualize the 5′ and 3′ ends of each of them. Because most piRNAs match multiple chromosomal sites, the software provides the number of occurrence inside the genome. Nonetheless, NucBase can restrict the analysis to reads that match the genome at a unique position. Based on release 5 of the Drosophila genome, 8,685 piRNAs displayed a unique position within *flam*. 235 of the unique piRNAs are encoded by the - strand and 8,450 are encoded by the + strand (Figure 2a). These data may then point to regulatory molecules encoded from piRNA clusters that may provide genomic function independently from transposable element (TE) regulation.

TE families are repeated within the genome, but they also display immobile vestiges with sequence mismatches. NucBase can sort out a sub-population of piRNAs that display a defined number of mismatches. When piRNAs homologous to TEs and encoded by *flam* were analyzed with two mismatches allowed, 134,040 piRNAs were identified with 90,444 piRNA produced from the + genomic strand and 43,596 from the - genomic strand of *flam*.

An option was designed to present alignments and highlight the unmatched nucleotides in target sequences. As exemplified for a fragment of *flamenco* (X: 21,503,826 to 21,504,465, Figure 2b) matched and unmatched sequences can be visualized as nucleotides and stars respectively along the target sequence.

Then, we used NucBase to identify the piRNAs homologous to ZAM transcripts (Figure 2c). The genomic population of piRNAs that displays a sequence complementary to mRNAs encoded by ZAM was extracted from Brennecke’s database. A total of 419 piRNAs were identified. To estimate how many of them are typically produced from the *flam* locus, these 419 piRNAs were compared to the *flam* sequence. NucBase sorted out 359 piRNA whose sequence is complementary and 100% identical to the ZAM mRNA. This result confirms that the major piRNA cluster regulating ZAM mobilization is indeed the *flam* locus, since only 60 piRNAs identical to ZAM are produced by other genomic loci.

## Conclusions

NucBase is an efficient and easy handling tool to extract sequences from large databases. It will be an invaluable help to biologists analyzing transposable element silencing by small non-coding RNAs. Although its capacities have been developed and tested for research concerning TE silencing and small RNA identification, there is no doubt that NucBase will also be useful to explore small nucleic sequences (DNA or RNA) in any organism.

## Availability and requirements

• Project name: NucBase

• Project home page: http://srv-gred.u-clermont1.fr/nucbase

• Operating system(s): Windows, Linux, Mac OS X 10.6

• Programming language: C++

• Other requirements: Qt and libdivsufsort (provided)

• License: LGPL

• Any restrictions to use by non-academics: The application in its current form may not be used for commercial purposes.

## Competing interests

The authors declare that they have no competing interests.

## Authors’ contributions

JD, PPo and CV conceived the software. PPo wrote the software. EB, JD and PPo realized the experiments and analyzed the data. JD, PPo, PPe, EB and CV wrote the manuscript. All authors read and approved the final manuscript.
